# Prevention of hospital-acquired pneumonia in non-ventilated adult patients: a narrative review

**DOI:** 10.1186/s13756-016-0150-3

**Published:** 2016-11-14

**Authors:** Leonor Pássaro, Stephan Harbarth, Caroline Landelle

**Affiliations:** 1 Infection Control Programme, Geneva University Hospitals and Medical School, Geneva, Switzerland; 2 Infection Control Unit, Centre Hospitalier Universitaire (CHU) Grenoble Alpes, CS 10217, 38043 Grenoble Cedex 9, France; 3 ThEMAS TIM-C UMR 5525, University Grenoble Alpes/CNRS, Grenoble, France

**Keywords:** Hospital-acquired pneumonia, Nosocomial pneumonia, Low respiratory tract infection, Prevention

## Abstract

**Background:**

Pneumonia is one of the leading hospital-acquired infections worldwide and has an important impact. Although preventive measures for ventilator-associated pneumonia (VAP) are well known, less is known about appropriate measures for prevention of hospital-acquired pneumonia (HAP).

**Aim:**

The purpose of this narrative review is to provide an overview of the current standards for preventing HAP in non-ventilated adult patients.

**Methods:**

A search of the literature up to May 2015 was conducted using Medline for guidelines published by national professional societies or professional medical associations. In addition, a comprehensive search for the following preventive measures was performed: hand hygiene, oral care, bed position, mobilization, diagnosis and treatment of dysphagia, aspiration prevention, viral infections and stress bleeding prophylaxis.

**Findings:**

Regarding international guidelines, several measures were recommended for VAP, whilst no specific recommendations for HAP prevention in non-ventilated patients are available. There is reasonable evidence available that oral care is associated with a reduction in HAP. Early mobilization interventions, swift diagnosis and treatment of dysphagia, and multimodal programmes for the prevention of nosocomial influenza cross-infection, have a positive impact on HAP reduction. The impact of bed position and stress bleeding prophylaxis remains uncertain. Systematic antibiotic prophylaxis for HAP prevention should be avoided.

**Conclusion:**

Scant literature and little guidance is available for the prevention of HAP among non-ventilated adult patients. In addition, the criteria used for the diagnosis of HAP and the populations targeted in the studies selected are heterogeneous. Oral care was the most studied measure and was commonly associated with a decrease in HAP rate, although a broad range of interventions are proposed. No robust evidence is available for other measures. Further high-quality studies are required to evaluate the impact of specific measures on HAP prevention in non-ventilated adult patients.

## Background

During the first European point prevalence survey (PPS) of healthcare-associated infections (HAI) and antimicrobial use in acute care hospitals, the most frequently reported HAI types were respiratory tract infections (pneumonia 19.4% and lower respiratory tract 4.1%) [1]. In 2013, the second PPS of HAI and antimicrobial use in European long-term care facilities also reported that the most frequently types of HAI were respiratory tract infections [2]. In 2005, the classification of nosocomial pneumonia was defined by the *American Thoracic Society* and the *Infectious Diseases Society of America* (ATS/IDSA) and sub-classified “hospital-acquired pneumonia” (HAP), “ventilator-associated pneumonia” (VAP) and “healthcare-associated pneumonia” (HCAP) [3]. HAP is defined as a pneumonia occurring 48 h or more after hospital admission and VAP refers to pneumonia occurring more than 48–72 h after endotracheal intubation. HCAP refers to pneumonia developing outside the hospital, amongst patients in close contact with the healthcare system, which shares epidemiologic, bacteriologic and pathogenic features with HAP [3, 4]. The incidence of HAP ranges from 5 to more than 20 cases per 1000 hospital admissions [3, 5]. Outside the intensive care unit (ICU), highest rates are observed in the elderly, immunocompromised hosts, surgical patients and those receiving enteral feeding through a nasogastric tube [3]. Almost one-third of HAPs are ICU-acquired, with VAP accounting for 90% of those cases. Overall, VAP occurs in 9–40% of intubated patients, with decreasing incidence rates during the last decade [6, 7].

Risk factors identified for HAP are older age, male sex, structural lung disease and multiorgan system failure [3]. Gram negative bacteria are the most frequent etiologic agents of HAP, namely *Pseudomonas aeruginosa*, *Klebsiella* spp, *Escherichia coli* and *Acinetobacter baumannii* [3, 8]. Among Gram positive pathogens, methicillin-resistant *Staphylococcus aureus* (MRSA) is the most frequent pathogen, particularly in immunocompromised patients [3]. Compared with community-acquired pneumonia, HAP are more likely to be infections caused by multidrug-resistant (MDR) pathogens [9]. The frequency of MDR pathogens as the etiologic agents of HAP and VAP is increasing [10, 11]. Factors, including antibiotic use or hospitalization in the previous 3 months, immunosuppression and exposure to specific hospital units with high frequency of antibiotic resistance, influence the likelihood of MDR pathogens infection [12].

Patients developing HAP are more likely to die, require intensive care, mechanical ventilation, and to have longer hospital length of stay (LOS) than patients without HAP [13]. Aspiration is one of the pathologic mechanisms involved in HAP and is associated with supine position, dysphagia, altered mental status, esophageal motility disorders, vomiting, gastroesophageal reflux, tracheal intubation, enteral nutrition, nasogastric intubation, oropharyngeal and tracheobronchial colonization, tracheostomy and alcoholism [3, 8, 14, 15].

Although preventive measures for VAP are well known, less is known about appropriate measures for prevention of HAP [16]. Hence, preventive measures directed to individuals without mechanical ventilation are of great importance for patient safety, as well as mitigating the potential costs of HAP. In this narrative review, we provide an overview of the current standards and lack of evidence-based guidance concerning specific preventive measures against HAP in non-ventilated adult patients.

## Literature search and selection strategy

A search for guidelines prior to May 2015 was performed using the electronic database Medline using the following terms: “pneumonia” AND (“nosocomial” OR “hospital-acquired”) AND “guidelines”. The results of this search were filtered to select for guidelines published by national professional societies or professional medical associations. An additional search was performed using Google Scholar and the Forum of European Respiratory Societies.

Medline was also used to search for articles published between January 2005 and May 2015 using the following terms: (“pneumonia” OR “respiratory tract infection” OR “respiratory infection”) AND (“nosocomial” OR “hospital-acquired” OR “healthcare associated” OR “healthcare-associated”) AND “prevention”. In addition, a review of each publications’ bibliography was performed to identify further pertinent articles.

For specific HAP prevention measures, a search using Medline for articles published prior to May 2015 was conducted using the following terms: (“pneumonia” OR “respiratory tract infection” OR “respiratory infection”) AND (“nosocomial” OR “hospital-acquired” OR “healthcare associated” OR “healthcare-associated”) AND:

- (“hand hygiene” OR “hand washing”)
- (“oral care” OR “oral hygiene” OR “oral decontamination” OR “oral health” OR “mouthwashes”)
- (“bed position” OR “head position” OR “body position” OR “bed rest” OR “bed protocol”)
- (“mobilization” OR “mobility” OR “motility” OR “physical activity” OR “physiotherapy”)
- (“dysphagia” OR “swallowing” OR “swallowing disorder”)
- (“aspiration” OR “aspirative”)
- (“viral infection” OR “viral” OR “virus” OR “flu”)
- (“stress bleeding prophylaxis” OR “gastric” OR “gastric protection” OR “acid-suppressive therapy” OR “acid-suppressive” OR “proton pump inhibitor” OR “omeprazol” OR “ranitidine”)

Articles published in English, French, Portuguese and Spanish were included in the review. Research was restricted to adult patients only. Studies performed on patients ventilated through a tracheal device (tracheal tube or tracheostomy) were excluded; studies performed on patients requiring non-invasive mechanical ventilation were included in the scope of the review. Studies which did not analyse the effect of any prevention measure were also excluded. Focus was placed on identifying the most relevant publications, namely meta-analyses, systematic reviews and studies containing results from randomized control trials (RCT) except for mobilization where all relevant studies were included. Some infection control bundle studies were also included when relevant.

## Professional societies’ guidelines and recommendations

There are several recommendations for pneumonia prevention issued by international guideline committees and professional societies. Although recommendations differ, all of the guidelines available focus on VAP prevention and not specifically on prevention of pneumonia outside the ICU (Table 1). General agreement was found for the following VAP preventive measures: hand hygiene, microbiologic monitoring, bed elevation, oral care with antiseptic, use of orogastric tubes, avoidance of endotracheal intubation and promotion of non-invasive ventilation [3, 8, 17–28]. Physiotherapy or mobilization as a preventive measure for VAP has not been equally addressed by the different societies. Among VAP prevention recommendations, some measures could theoretically also target HAP prevention, such as hand hygiene, oral care with antiseptic, aspiration prevention, bed elevation and early mobilization.

**Table 1:** Guidelines and recommendations for the management of hospital-acquired pneumonia, ventilator-associated pneumonia and healthcare-associated pneumonia

Author/Society	Country/Region	Year	HAP prevention measures	VAP prevention measures	Hand hygiene	Microbiologic monitoring	Invasive device removal	Avoid endotracheal intubation	Reduction in the prescription of AB
Centres for Disease Control and Prevention [17]	USA	2003	NA	Available	R	R	DR	R	DR
Infectious Diseases Society of America [3]	USA	2005	NA	Available	R	R	DR	R	DR
Latin American Thoracic Society [18]	Latin America	2005	NA	Available	R	R	DR	R	DR
Federation of the Infectious Diseases Societies of South Africa [19]	South Africa	2005	NA	Available	R	R	DR	DR	R
Association for Professionals in Infection Control and Epidemiology [20]	USA	2005	NA	Available	R	R	R	R	R
The South African Thoracic Society [21]	South Africa	2006	NA	Available	R	R	R	R	R
Brazilian Society of Pneumology and Tisiology [22]	Brazil	2007	NA	Available	R	R	R	R	R
Portuguese Societies of Pneumology and Intensive Care [23]	Portugal	2007	NA	Available	R	R	R	R	R
The British Society for Antimicrobial Therapy [24]	England	2008	NA	Available	R	R	DR	R	DR
Association of Medical Microbiology and Infectious Diseases [25]	Canada	2008	NA	Available	R	R	R	R	R
European Society of Clinical Microbiology and Infectious Diseases [8]	Europe	2009	NA	Available	R	R	R	R	R
The French Society of Hospital Hygiene [26]	France	2010	NA	Available	R	DR	DR	R	DR
The Spanish Society of Pulmonology and Thoracic Surgery [27]	Spain	2011	NA	Available	R	R	R	R	R
Singapore Infection Control Association and Singapore Society of Intensive Care Medicine [28]	Singapore	2013	NA	Available	R	R	DR	DR	DR
Author/Society	Avoid reintubation	Promote NIV if possible	Orogastric tubes	Cuff pressure (mmHg)	Bed elevation	Subglotic aspiration	Silver or AB endotracheal tube coating	Oral decontamination	SDD
Centres for Disease Control and Prevention [17]	R	R	R	DR	R	NR	DR	NR	NR
Infectious Diseases Society of America [3]	R	R	R	20	R	R	DR	NR	NR
Latin American Thoracic Society [18]	R	R	R	DR	R	R	DR	R	NR
Federation of the Infectious Diseases Societies of South Africa [19]	DR	DR	R	DR	R	NR	DR	R	NR
Association for Professionals in Infection Control and Epidemiology [20]	DR	R	DR	DR	R	R	DR	R	NR
The South African Thoracic Society [21]	DR	DR	R	DR	R	DR	DR	DR	DR
Brazilian Society of Pneumology and Tisiology [22]	NR	R	DR	20	R	R	DR	R	NR
Portuguese Societies of Pneumology and Intensive Care [23]	R	R	R	DR	R	DR	DR	R	DR
The British Society for Antimicrobial Therapy [24]	R	R	DR	25	R	R	DR	DR	R
Association of Medical Microbiology and Infectious Diseases [25]	R	R	R	20	R	R	DR	R	NR
European Society of Clinical Microbiology and Infectious Diseases [8]	R	R	R	20	R	R	DR	R	NR
The French Society of Hospital Hygiene [26]	DR	R	R	25-30	R	NR	DR	R	NR
The Spanish Society of Pulmonology and Thoracic Surgery [27]	DR	R	DR	DR	R	R	R	R	NR
Singapore Infection Control Association and Singapore Society of Intensive Care Medicine [28]	DR	DR	R	DR	R	R	DR	R	DR

*AB* antibiotic, *DR* don’t refer, *HAP* hospital-acquired pneumonia, *NA* not available, *R* recommended, *VAP* ventilator-associated pneumonia, *NIV* non-invasive ventilation, *NR* not recommended, *SDD* selective digestive decontamination

## Measures for HAP prevention

The heterogeneity of the interventions investigated (hand hygiene, oral care, prevention of aspiration and dysphagia, bed position, mobilization, prevention of viral infections, antibioprophylaxis and stress-bleeding prophylaxis) did not permit a meta-analysis.

### Hand hygiene

Hand hygiene is an effective measure to prevent HAI [29–32]. Nevertheless, no clinical trial has demonstrated its efficacy for decreasing specifically pneumonia outside the ICU. Pittet et al. [29] showed that access to bedside antiseptic handrubs contributed to an increase in hand hygiene compliance leading to an overall significant reduction in nosocomial infection prevalence (from 16.9 to 9.9%). Hence, the implementation of programmes to enhance hand hygiene adherence by health care workers (HCWs) and use of alcohol-based disinfectants could potentially contribute to HAP reduction, but further studies are needed to demonstrate its preventive effectiveness, independent of other measures.

### Oral care

Aspiration of oropharyngeal secretions is an important pathogenic event preceding HAP. Silent aspiration into the intrathoracic airway occurs in normal subjects and is more pronounced in elderly and neurologically impaired patients [15, 33]. The impact of oral care in reducing respiratory tract infections, HAP and mortality has been documented in several clinical trials and has been subject to systematic reviews and meta-analysis [34–37]. Three systematic reviews and one meta-analysis addressing the preventive effect of oral hygiene in pneumonia among individuals staying at health-care facilities were selected (Table 2).

**Table 2:** Systematic reviews about the association between oral care and reduction in pneumonia or respiratory tract infection

Author, journal, date	Type of studies included	Population	Type of intervention	Major conclusion
Azarpazhooh A. et al., J Periodontol, 2006 [37]	Systematic Review (7 RCT, 3 control trials)	Elderly adults living in nursing homes, ICU patients and patients from a general hospital	Professional dental care, mouthrinse with 0.12 % CHX, application of 0.2% CHX gel, 1% PVI scrubbing of pharynx, topical application of a non-absorbable antibiotic solution and topical antimicrobial prophylaxis	Oral care reduces incidence of pneumonia
Sjorgen P. et al., J Am Geriatr Soc, 2008 [36]	Systematic Review (5 RCT, 1 systematic review, 3 case–control trials, 5 cross-sectional trials, 1 retrospective trial)	Dependent elderly people	0.12% CHX oral rinse, tooth-brushing, 1% PVI scrubbing of pharynx or professional mechanical oral health care weekly	Oral care prevents pneumonia, respiratory tract infection and death from pneumonia
Kaneoka A. et al., Infect Control Hosp Epidemiol, 2015 [34]	Systematic Review (4 RCT) and Meta-analysis (5 RCT)	Hospitalized non-ventilated patients and patients living in nursing homes	0.2% CHX application, 0.12% CHX gargle, tooth brushing performed by dental professionals, ± 1% PVI scrubbing of pharynx	Oral care prevents pneumonia and fatal pneumonia in non-ventilated patients
El-Rabbany M. et al., Int J Nurs Stud, 2015 [35]	Systematic Review (28 RCT)	Elderly adults living in nursing homes and ICU patients	Professional dental care, sodium bicarbonate mouthrinse, toothbrushing, 0.12% and 0.2% CHX, topical application of a non-absorbable antibiotic solution and PVI swab	Oral care was suggested to be associated with a reduction in the risk for HAP and VAP in high-risk patients

*CHX* chlorhexidine, *HAP* hospital-acquired pneumonia, *ICU* Intensive Care Unit, *PVI* povidone-iodine, *RCT* Randomized Control Trials, *VAP* ventilator-associated pneumonia

Azarpazhooh et al. [37] conducted a systematic review to examine the evidence for a possible etiological association between oral health and pneumonia. The review found that presence of cariogenic and periodontal pathogens in saliva and dental plaque, dental decay and poor oral hygiene were potential risk factors for HAP. Ten studies analysing the impact of oral care interventions in the incidence or progression of pneumonia were analysed; three of them were not randomized. The interventions included in the studies were: professional dental care, mouthrinse with 0.12% chlorhexidine (CHX), application of 0.2% CHX gel, 1% povidone-iodine (PVI) scrubbing of pharynx, topical application of a non-absorbable antibiotic solution and topical antimicrobial prophylaxis. Studies were performed in ICUs (*n* = 6), nursing homes (*n* = 3) and in a general hospital (*n* = 1). Except for 1 study, all studies showed that interventions reduced the incidence of pneumonia and/or the length of mechanical ventilation. Overall, this review found a relative risk reduction in pneumonia incidence between 34 and 83% following oral decontamination techniques.

Sjorgen et al. [36] conducted a systematic review focusing on the preventive attributes of oral hygiene (0.12% CHX oral rinse, tooth-brushing, 1% PVI scrubbing of pharynx or professional mechanical oral health care weekly) on pneumonia and respiratory tract infection among hospitalized elderly people and elderly nursing home residents. It included five RCTs and 10 non-randomized intervention studies, all suggesting an association between poor oral hygiene and pneumonia in dependent elderly people. Data from the included RCTs were not considered for meta-analysis because of the heterogeneity in primary endpoints, methodological quality and study design. The analysis revealed an absolute risk reduction between 6.6 and 11.7% for pneumonia, respiratory tract infection and death from pneumonia.

A meta-analysis investigating the effect of oral care on pneumonia among non-ventilated patients was conducted by Kaneoka et al. and included five RCTs, two of which assessed the use of CHX in hospitalized patients and the remaining three the impact of mechanical oral cleaning among nursing home residents [34]. A significant risk reduction for pneumonia (relative risk (RR) 0.61, 95% CI 0.40–0.91) as well as a risk reduction for fatal pneumonia (RR 0.41, 95% CI 0.23–0.71) were observed. However, these results should be interpreted with caution. The majority of the RCTs were at high risk of selection bias in that they did not include participants with risk factors for pneumonia, such as patients with nasogastric tubes or severe dementia. Furthermore, a precise and reproducible definition of pneumonia was not provided in two RCTs.

El-Rabbany et al. [35] updated the Azarpazhoo et al. systematic review in 2015, including the only RCTs which evaluated the impact of different health care strategies on incidence of HAP and VAP. Twenty-eight RCTs were selected for analysis and addressed the following interventions: 1) professional dental care, 2) sodium bicarbonate mouthrinse, 3) toothbrushing, 4) 0.12 and 0.2% CHX, 5) topical application of a non-absorbable antibiotic solution and 6) PVI swab. Although all included trials had a randomized controlled design, the risk-of-bias evaluation revealed that the majority of the included studies presented with a moderate to high risk of bias [38]. Overall, the use of oropharyngeal decontamination using various antimicrobial interventions was suggested to be associated with a reduction in both VAP and HAP. Twelve of the 17 studies reviewing the efficacy of CHX failed to demonstrate a significant effect. The effectiveness of other measures such as tooth brushing or iodine swab remained uncertain.

### Prevention of aspiration and dysphagia

Dysphagia is the most important risk factor for aspiration pneumonia, especially in elderly and acute stroke patients. It is estimated that 43–54% of stroke patients with dysphagia aspirate and 37% of the later will develop pneumonia [39].

A systematic review published in 2001 including 1808 studies analysed dysphagia programmes and prevention of pneumonia among post-stroke patients, mainly focusing on the methodology used for the diagnosis of dysphagia [39]. The diagnostic methods analysed were: 1) patients’reports of swallowing difficulty, 2) bedside program evaluation, 3) videofluoroscopic study of swallowing and 4) fiberoptic endoscopic examination of swallowing. Although no RCTs were found in the search, the implementation of a systematic programme for diagnosis and treatment of dysphagia in acute stroke patients seemed to substantially reduce pneumonia rates compared with historical controls in 4 different case series. The small size of available studies did not allow determination of the relative efficacy of different diagnostic methods.

Foley et al. conducted in 2008 a systematic review including 15 RCTs that analysed the treatment of dysphagia in post-stroke patients in respect of death, return to functional swallowing and pneumonia [40]. The treatments analysed in the review were: 1) texture-modified diets, 2) swallowing therapy programmes, 3) non-oral feeding, 4) use of medications (nifedipine) and 5) physical stimulation (aromatherapy, cold stimulus of the faucial pillars). According to the authors, the methodological quality of the trials was only fair. Due to the small number of trials found in the search as well as the heterogeneity of treatments and outcomes evaluated, limited evidence was found to support a specific treatment for dysphagia. Nevertheless, the analysis performed suggested that nasogastric tubes do not appear to increase the risk of death when compared with percutaneous endoscopic gastrostomy feeding tubes. Furthermore, general swallowing programs were associated with a pneumonia risk reduction in acute stroke patients.

### Bed position

The positioning of mechanically ventilated patients in a semi-recumbent position for preventing pneumonia has been advocated for more than a decade [3, 8, 17–28]. However, the possible impact of semi-recumbent position in HAP prevention among non-ventilated patients has not been extensively studied.

In 1992, a RCT including 45 dependent patients admitted to a Japanese geriatric hospital analysed the impact of bed elevation for at least two hours after each meal and daily cleaning of the oropharynx (gargling for a few minutes with PVI) compared to standard care in the prevention of respiratory tract infections [41]. Febrile days were significantly decreased (up to 4 days) in the intervention group compared to the control group. However, which measure (oral hygiene or bed elevation) contributed more to the reduction in febrile days could not be assessed in this study.

Loan et al. conducted a RCT including 229 adults and children with tetanus admitted to a Vietnamese hospital [42]. The intervention group was assigned to a semi-recumbent position of 30°. The study included ventilated and non-ventilated patients as well as tracheostomized subjects. Development of pneumonia and mortality did not differ between the intervention (semi-recumbent position) and control group (supine position). In fact, an increase in the overall complication rate (65.0% versus 50.9%, *p* = 0.03) and a need for tracheostomy (58.9% versus 45.5%, *p* = 0.04) were observed in the intervention group. Thus, clinicians should be aware of the risk that non-ventilated patients may often change their positioning and may even increase the likelihood of microaspirations and further complications.

### Mobilization

Development of HAP is associated with a reduction in respiratory secretions’ clearance, which in turn is related to physical inactivity. Some recommendations for VAP prevention include physiotherapy and early mobility programmes [3], but sparse data is available for HAP prevention.

Cuesy et al. conducted a RCT evaluating the preventive effect of a “turn-mob” program for HAP [43]. The study included 223 non-ventilated patients with acute ischemic stroke. The intervention group (*n* = 111) was submitted to the “turn-mob” program, the later consisting of modifying the patient from supine to right and left lateral recumbent position every two hours and passive mobilization of the limbs every 6 h. The intervention was carried out by previously trained relatives of the patient. The control group (*n* = 112) was submitted to change of position by the nursing staff 3 times per day. Performing passive mobilization and postural changes was associated with a 61% relative decrease in the incidence of HAP (intervention group- 12.6% versus control group- 26.8%; RR 0.39, 95% CI 0.19–0.79). Of note, the high HAP incidence (19.7%) observed in this study is twice the rate usually reported for acute stroke patients [44]. Furthermore, the turn-mob program was performed every two hours; thus, wide implementation of this strategy, carried out by patient family members or by HCWs, seems to be too cumbersome and time-consuming to be applied broadly.

A prospective cluster study including 1179 subjects from elderly and respiratory care compared the effect of an early mobility bundle programme in one hospital to usual care in a second one [45]. The primary outcome was incidence of HAP. The intervention consisted of enhancing measures and equipment (availability of walking aids, of mobility charts, education…) in order to maximize patients’mobility. After adjustement on admission condition, age and patient comorbidity, the intervention remained associated with lower incidence of HAP with a hazard ratio of 0.39 (95% CI 0.22–0.68). But these results should be interpreted with caution: patients were not randomized and there were some significant differences between the groups in terms of demographics and comorbidities; falls were significantly higher in the intervention group in comparison with the control group (29.2% versus 18.4%). Randomized studies are warranted to prove clinical effectiveness and lack of adverse events of this kind of intervention for elderly patients.

### Other preventive interventions

Besides the above mentioned interventions, other preventive measures have been considered for the prevention of HAP.

*Viral infections*- among patients with HAP, approximately 20% are due to viral pathogens and are associated with increased morbidity and mortality [46, 47]. Respiratory viral infections, especially influenza and respiratory syncytial virus, affect mainly immunocompromised patients and nosocomial transmission is common [48]. Multimodal interventions including 1) contact and droplet precautions, 2) cohort nursing, 3) influenza vaccination of HCW and high risk populations, 4) chemoprophylaxis to residents in long term care facilities during an influenza outbreak and 5) generalized use of masks irrespective of vaccination status have shown to be effective in prevention of nosocomial spread of influenza and other respiratory viruses [46, 48–50].

*Antibioprophylaxis*- acute stroke patients are especially vulnerable to infections due to diverse factors: swallowing disturbances, altered mental status, use of invasive procedures (urinary catheterisation; mechanical ventilation) and immunodepression [51]. Considering the higher risk of infection among these patients, a possible benefit by preemptive antibiotic therapy has been considered. A Cochrane systematic review found six RCTs including 506 acute ischemic or haemorrhagic stroke patients. Of these, five were included in a meta-analysis and identified a significant reduction in the general infection rate from 36 to 22% with antibiotic prophylaxis [51]. A recent multicentre RCT included 2538 patients with acute stroke to investigate the effect of intravenous ceftriaxone (2 g daily for 4 days) in the functional outcome at 3 months (modified Rankin Scale), infection rates, death, antimicrobial use and LOS [52]. Although the intervention group had a significant reduction in the rates of overall infection (odds ratio (OR) 0.44, 95% CI 0.30–0.65), the same effect was not observed for pneumonia (OR 0.67, 95% CI 0.39–1.15). Neither functional outcome, nor mortality or LOS was reduced with the intervention, and therefore, there is currently not enough evidence of benefit from the use of prophylactic antibiotics in acute stroke patients.

*Stress-bleeding prophylaxis*- increase in gastric pH can lead to an increase in bacterial colonization. Sultan et al. [53] have performed a meta-analysis to examine the association between proton pump inhibitors (PPIs) treatment and respiratory infections. They included 7 RCTs and showed a trend towards an association between PPIs and respiratory infections, although it failed to reach statistical significance (OR 1.42, 95% CI 0.86–2.35; *P* = 0.17). Eom et al. [54] have studied the association between the use of acid-suppressive drugs and the risk of pneumonia. Meta-analysis of 23 RCTs examining risk of HAP in association with use of histamine-2 receptor antagonists showed a higher risk of HAP among subjects receiving those drugs (RR 1.22, 95% CI 1.01–1.48). Of note, few RCTs have been performed outside the ICU setting.

*Multimodal strategies*- Juthani-Metha et al. conducted a cluster RCT evaluating the impact of a multimodal intervention protocol for pneumonia prevention among 834 elderly people residing in 36 nursing homes [55]. The study was limited to patients at high risk for pneumonia (residing at a nursing home for at least 1 month, and who had at least one of two risk factors for pneumonia: impaired oral hygiene; swallowing difficulty) which does not represent the entire elderly population residing in nursing homes [56]. The intervention consisted of manual tooth/gum brushing and 0.12% CHX oral rinse twice daily, plus maintaining an upright position during feeding. The intervention did not reduce the incidence of pneumonia nor respiratory tract infections compared to usual care. Of note, the trial was terminated early for futility.

## Conclusion

Data concerning prevention of HAP in non-ventilated adult patients, breathing without assistance through a tracheal tube is scarce and of a poor quality. Therefore formal recommendations and evidence-based guidelines are not available. This is in contrast to VAP prevention that encompasses a large body of evidence and important research efforts, leading to strong guidelines and recommendations from professional societies.

Regarding oral care, the majority of studies included in reviews and meta-analyses had a high risk of bias and there was heterogeneity concerning the type of interventions studied. Nevertheless, chemical oral care is associated with a reduction in HAP and mortality [35–37]. Further studies are needed to clarify the impact of specific measures (CHX, toothbrush, PVI and professional oral care), their optimal frequency, and whether there is an existence of a synergistic combination of different oral care interventions on HAP prevention.

Implementation of systematic programs for the diagnosis and early treatment of dysphagia may lead to dramatic reductions in HAP rates among neurologically impaired patients [39, 40]. Further studies are needed to define the best diagnostic approach as well as the best treatment modality for dysphagia.

Implementation of multimodal programs for the prevention of nosocomial transmission of viral infections, especially seasonal and pandemic influenza is a cornerstone of HAP prevention during winter season [49]. Universal use of mask irrespective of vaccination status is effective in reducing nosocomial transmission of influenza, but may be difficult to implement [57, 58]. HCW vaccination, cohort nursing and antiviral prophylaxis for high risk patients during an outbreak reduce transmission and the deleterious consequences of influenza infection, including lower respiratory tract infections [46, 48].

Although only two studies were found, early mobilization interventions appear to reduce HAP incidence [45]. Post-stroke patients in particular could benefit from early mobilization, especially in the 48 h after the ischemic event [43]. But the benefit/risk balance of intensive programs needs to be further evaluated, monitoring also the risk of falls.

We were unable to make definite conclusions regarding the impact of bed position or stress bleeding prophylaxis on HAP due to the small number of studies performed as well conflicting results between them. Systematic antibioprophylaxis for HAP prevention should be avoided.

Although no clinical trial has demonstrated efficacy of hand hygiene for decreasing specifically HAP, standard precautions and cleaning practices are the cornerstone to reduce cross-infection. Focusing prevention on reservoirs, with good respect of hand hygiene, and the portal of entry, by improving basic oral care, may be the most realistic approaches for preventing HAP in non-ventilated patients.

A number of limitations should be kept in mind regarding the available evidence. Firstly, the criteria used for HAP diagnosis were heterogeneous across the literature. Second, few RCTs were found concerning most preventive measures, and some studies had small sample sizes. Third, the populations targeted in the studies, the interventions and the outcomes analysed were very heterogeneous.

In conclusion, for clinical practice, the following measures may be recommended for non-intubated adult patients despite the above-cited methodological limitations: 1) to respect the five moments for hand hygiene [59], 2) to perform daily oral care with antiseptics, 3) to implement a systematic programme for diagnosis and treatment of dysphagia among post-stroke patients, 4) to implement interventions for preventing the nosocomial spread of viral infections during winter season, 5) to support early mobilization of patients by taking into account the risk of falls, 6) to avoid systematic antibiotic prophylaxis for HAP prevention. The effect of bed position, acid-suppressive drugs as well as the synergistic effect of more than one preventive measure on HAP incidence needs to be evaluated in further controlled trials.

## Abbreviations

ATS/IDSA: American Thoracic Society / Infectious Diseases Society of America
CHX: Chlorhexidine
HAI: Healthcare-associated infections
HAP: Hospital-acquired pneumonia
HCAP: Healthcare-associated pneumonia
HCWs: Health care workers
ICU: Intensive care unit
LOS: Length of stay
MDR: Multidrug-resistant
MRSA: Methicillin-resistant *Staphylococcus aureus*
OR: Odds ratio
PPIs: Proton pump inhibitors
PPS: Point prevalence survey
PVI: Povidone-iodine
RCT: Randomized control trials
RR: Relative risk
VAP: Ventilator-associated pneumonia

## References

[1] European Centre for Disease Prevention and Control (2013). Point prevalence survey of healthcare associated infections and antimicrobial use in European acute care hospitals.

[2] European Centre for Disease Prevention and Control (2014). Point prevalence survey of healthcare associated infections and antimicrobial use in European long-term care facilities.

[3] American Thoracic Society, Infectious Diseases Society of America (2005). Guidelines for the management of adults with hospital-acquired, ventilator-associated, and healthcare-associated pneumonia. Am J Respir Crit Care Med.

[4] Scholte JB, van Mook WN, Linssen CF (2014). Surveillance cultures in healthcare-associated pneumonia: sense or nonsense?. Curr Opin Pulm Med.

[5] Chawla R (2008). Epidemiology, etiology, and diagnosis of hospital-acquired pneumonia and ventilator-associated pneumonia in Asian countries. Am J Infect Control.

[6] Vincent JL, Rello J, Marshall J, Silva E, Anzueto A, Martin CD (2009). International study of the prevalence and outcomes of infection in intensive care units. JAMA.

[7] Rosenthal VD, Bijie H, Maki DG, Mehta Y, Apisarnthanarak A, Medeiros EA (2012). International Nosocomial Infection Control Consortium (INICC) report, data summary of 36 countries, for 2004–2009. Am J Infect Control.

[8] Torres A, Ewig S, Lode H, Carlet J, European HAP working group (2009). Defining, treating and preventing hospital acquired pneumonia: European perspective. Intensive Care Med.

[9] Cardoso T, Almeida M, Carratalà J, Aragão I, Costa-Pereira A, Sarmento AE (2015). Microbiology of healthcare-associated infections and the definition accuracy to predict infection by potentially drug resistant pathogens: a systematic review. BMC Infect Dis.

[10] Denys GA, Relich RF (2014). Antibiotic resistance in nosocomial respiratory infections. Clin Lab Med.

[11] Barbier F, Andremont A, Wolff M, Bouadma L (2013). Hospital-acquired pneumonia and ventilator-associated pneumonia: recent advances in epidemiology and management. Curr Opin Pulm Med.

[12] Sydnor ER, Perl TM (2011). Hospital epidemiology and infection control in acute-care settings. Clin Microbiol Rev.

[13] Micek ST, Chew B, Hampton N, Kollef MH. A case–control study assessing the impact of non-ventilated hospital- acquired pneumonia on patient outcomes. Chest. 2016;150(5):1008–14. 10.1016/j.chest.2016.04.009 PMC7094544 27102181

[14] Lomotan JR, George SS, Brandstetter RD (1997). Aspiration pneumonia. Strategies for early recognition and prevention. Postgrad Med.

[15] DiBardino DM, Wunderink RG (2015). Aspiration pneumonia: a review of modern trends. J Crit Care.

[16] Klompas M (2016). Hospital-acquired pneumonia in non ventilated patients: the next frontier. Infect Control Hosp Epidemiol.

[17] Healthcare Infection Control Practices Advisory Committee, Centers for Disease Control and Prevention (U.S.) (2004). Guidelines for preventing health-care-associated pneumonia, 2003 recommendations of the CDC and the Healthcare Infection Control Practices Advisory Committee. Respir Care.

[18] Luna CM, Monteverde A, Rodríguez A, Apezteguia C, Zabert G, Ilutovich S, Menga G, Vasen W, Díez AR, Mera J, Grupo Argentino-Latino Americano de estudio de la Neumonía Nosocomial (GALANN) (2005). Clinical guidelines for the treatment of nosocomial pneumonia in Latin America: an interdisciplinary consensus document. Recommendations of the Latin American Thoracic Society. Arch Bronconeumol.

[19] Dusé A (2005). Infection control in developing countries with particular emphasis on South Africa. S Afr J Epidemiol Infect.

[20] Flanders SA, Collard HR, Saint S (2006). Nosocomial pneumonia: state of the science. Am J Infect Control.

[21] Brink A, Feldman C, Duse A, Gopalan D, Grolman D, Mer M, Naicker S, Paget G, Perovic O, Richards G, South African Thoracic society (SATS) (2006). Guideline for the management of nosocomial infections in South Africa. S Afr Med J.

[22] Sociedade Brasileira de Pneumologia e Tisiologia (2007). Brazilian guidelines for treatment of hospital acquired pneumonia and ventilator associated pneumonia- 2007. J Bras Pneumol.

[23] Oliveira AG (2005). Current management of hospitalized community acquired pneumonia in Portugal. Consensus statements of an expert panel. Rev Port Pneumol.

[24] Masterton RG, Galloway A, French G, Street M, Armstrong J, Brown E, Cleverley J, Dilworth P, Fry C, Gascoigne AD, Knox A, Nathwani D, Spencer R, Wilcox M (2008). Guidelines for the management of hospital-acquired pneumonia in the UK: report of the working party on hospital-acquired pneumonia of the British Society for Antimicrobial Chemotherapy. J Antimicrob Chemother.

[25] Rotstein C, Evans G, Born A, Grossman R, Light RB, Magder S, McTaggart B, Weiss K, Zhanel GG (2008). Clinical practice guidelines for hospital-acquired pneumonia and ventilator-associated pneumonia in adults. Can J Infect Dis Med Microbiol.

[26] Société Française d'Hygiène Hospitalière. Surveiller et prévenir les infections associées aux soins. https://sf2h.net/publications/surveiller-prevenir-infections-associees-aux-soins. Accessed 11 Nov 2016.

[27] Blanquer J, Aspa J, Anzueto A, Ferrer M, Gallego M, Rajas O, Rello J, Rodríguez de Castro F, Torres A, Sociedad Española de Neumología y Cirugía Torácica (2011). SEPAR guidelines for nosocomial pneumonia. Arch Bronconeumol.

[28] Infection Control Association (Singapore), Society of Intensive Care Medicine (Singapore) (2013). Guidelines for the prevention of ventilator associated pneumonia.

[29] Pittet D, Hugonnet S, Harbarth S, Mourouga P, Sauvan V, Touveneau S, Perneger TV (2000). Effectiveness of a hospital-wide programme to improve compliance with hand hygiene. Infection Control Programme. Lancet.

[30] Marra AR, Guastelli LR, de Araújo CM, dos Santos JL, Lamblet LC, Silva M, de Lima G, Cal RG, Paes AT, Cendoroglo Neto M, Barbosa L, Edmond MB, dos Santos OF (2010). Positive deviance: a new strategy for improving hand hygiene compliance. Infect Control Hosp Epidemiol.

[31] Koff MD, Corwin HL, Beach ML, Surgenor SD, Loftus RW (2011). Reduction in ventilator associated pneumonia in a mixed intensive care unit after initiation of a novel hand hygiene program. J Crit Care.

[32] Boyce JM, Pittet D, Healthcare Infection Control Practices Advisory Committee, Society for Healthcare Epidemiology of America, Association for Professionals in Infection Control, Infectious Diseases Society of America. Hand Hygiene Task Force (2002). Guideline for Hand Hygiene in Health-Care Settings: recommendations of the Healthcare Infection Control Practices Advisory Committee and the HICPAC/SHEA/APIC/IDSA Hand Hygiene Task Force. Infect Control Hosp Epidemiol.

[33] Gleeson K, Eggli DF, Maxwell SL (1997). Quantitative aspiration during sleep in normal subjects. Chest.

[34] Kaneoka A, Pisegna JM, Miloro KV, Lo M, Saito H, Riquelme LF, LaValley MP, Langmore SE (2015). Prevention of healthcare-associated pneumonia with oral care in individuals without mechanical ventilation: a systematic review and meta-analysis of randomized controlled trials. Infect Control Hosp Epidemiol.

[35] El-Rabbany M, Zaghlol N, Bhandari M, Azarpazhooh A (2015). Prophylactic oral health procedures to prevent hospital-acquired and ventilator-associated pneumonia: a systematic review. Int J Nurs Stud.

[36] Sjögren P, Nilsson E, Forsell M, Johansson O, Hoogstraate J (2008). A systematic review of the preventive effect of oral hygiene on pneumonia and respiratory tract infection in elderly people in hospitals and nursing homes: effect estimates and methodological quality of randomized controlled trials. J Am Geriatr Soc.

[37] Azarpazhooh A, Leake JL (2006). Systematic review of the association between respiratory diseases and oral health. J Periodontol.

[38] Higgins JP, Altman DG, Gøtzsche PC, Jüni P, Moher D, Oxman AD, Savovic J, Schulz KF, Weeks L, Sterne JA, Cochrane Bias Methods Group, Cochrane Statistical Methods Group (2011). The Cochrane Collaboration’s tool for assessing risk of bias in randomised trials. BMJ..

[39] Doggett DL, Tappe KA, Mitchell MD, Chapell R, Coates V, Turkelson CM (2001). Prevention of pneumonia in elderly stroke patients by systematic diagnosis and treatment of dysphagia: an evidence-based comprehensive analysis of the literature. Dysphagia.

[40] Foley N, Teasell R, Salter K, Kruger E, Martino R (2008). Dysphagia treatment post stroke: a systematic review of randomised controlled trials. Age Ageing.

[41] Meguro K, Yamagauchi S, Doi C, Nakamura T, Sekizawa K, Sasaki H (1992). Prevention of respiratory infections in elderly bed-bound nursing home patients. Tohoku J Exp Med.

[42] Loan HT, Parry J, Nga NT, Yen LM, Binh NT, Thuy TT, Duong NM, Campbell JI, Thwaites L, Farrar JJ, Parry CM (2012). Semi-recumbent body position fails to prevent healthcare-associated pneumonia in Vietnamese patients with severe tetanus. Trans R Soc Trop Med Hyg.

[43] Cuesy PG, Sotomayor PL, Pina JO (2010). Reduction in the incidence of poststroke nosocomial pneumonia by using the “turn-mob” program. J Stroke Cerebrovasc Dis.

[44] Westendorp WF, Nederkoorn PJ, Vermeij JD, Dijkgraaf MG, van de Beek D (2011). Post-stroke infection: a systematic review and meta-analysis. BMC Neurol.

[45] Stolbrink M, McGowan L, Saman H, Nguyen T, Knightly R, Sharpe J, Reilly H, Jones S, Turner AM (2014). The Early Mobility Bundle: a simple enhancement of therapy which may reduce incidence of hospital-acquired pneumonia and length of hospital stay. J Hosp Infect.

[46] Goins WP, Talbot HK, Talbot TR (2011). Health care-acquired viral respiratory diseases. Infect Dis Clin North Am.

[47] Pagani L, Thomas Y, Huttner B, Sauvan V, Notaridis G, Kaiser L, Iten A, Pittet D, Harbarth S (2015). Transmission and effect of multiple clusters of seasonal influenza in a Swiss geriatric hospital. J Am Geriatr Soc.

[48] Raad I, Abbas J, Whimbey E (1997). Infection control of nosocomial respiratory viral disease in the immunocompromised host. Am J Med.

[49] Cheng VC, Tai JW, Wong LM, Chan JF, Li IW, To KK, Hung IF, Chan KH, Ho PL, Yuen KY (2010). Prevention of nosocomial transmission of swine-origin pandemic influenza virus A/H1N1 by infection control bundle. J Hosp Infect.

[50] Buchbinder N, Dumesnil C, Pinquier D, Merle V, Filhon B, Schneider P, Vannier JP (2011). Pandemic A/H1N1/2009 influenza in a paediatric haematology and oncology unit: successful management of a sudden outbreak. J Hosp Infect.

[51] Westendorp WF, Vermeij JD, Vermeij F, Den Hertog HM, Dippel DW, van de Beek D, Nederkoorn PJ (2012). Antibiotic therapy for preventing infections in patients with acute stroke. Cochrane Database Syst Rev.

[52] Westendorp WF, Vermeij JD, Zock E, Hooijenga IJ, Kruyt ND, Bosboom HJ, Kwa VI, Weisfelt M, Remmers MJ, ten Houten R, Schreuder AH, Vermeer SE, van Dijk EJ, Dippel DW, Dijkgraaf MG, Spanjaard L, Vermeulen M, van der Poll T, Prins JM, Vermeij FH, Roos YB, Kleyweg RP, Kerkhoff H, Brouwer MC, Zwinderman AH, van de Beek D, Nederkoorn PJ, PASS investigators (2015). The Preventive Antibiotics in Stroke Study (PASS): a pragmatic randomised open-label masked endpoint clinical trial. Lancet.

[53] Sultan N, Nazareno J, Gregor J (2008). Association between proton pump inhibitors and respiratory infections: a systematic review and meta-analysis of clinical trials. Can J Gastroenterol.

[54] Eom CS, Jeon CY, Lim JW, Cho EG, Park SM, Lee KS (2011). Use of acid-suppressive drugs and risk of pneumonia: a systematic review and meta-analysis. CMAJ.

[55] Juthani-Mehta M, Van Ness PH, McGloin J, Argraves S, Chen S, Charpentier P, Miller L, Williams K, Wall D, Baker D, Tinetti M, Peduzzi P, Quagliarello VJ (2015). A cluster-randomized controlled trial of a multicomponent intervention protocol for pneumonia prevention among nursing home elders. Clin Infect Dis.

[56] Mody L (2015). Editorial commentary: preventing aspiration pneumonia in high-risk nursing home residents: role of chlorhexidine-based oral care questioned again. Clin Infect Dis.

[57] MacIntyre CR, Chughtai AA (2015). Facemasks for the prevention of infection in healthcare and community settings. BMJ.

[58] Brandt C, Rabenau HF, Wicker S (2011). Attitudes of influenza-vaccinated health care workers toward masks to prevent nosocomial transmission of influenza. Influenza Other Respir Viruses.

[59] Sax H, Allegranzi B, Uçkay I, Larson E, Boyce J, Pittet D (2007). ‘My five moments for hand hygiene’: a user-centred design approach to understand, train, monitor and report hand hygiene. J Hosp Infect.

